# PSO-Transformer for mapping soil heavy metals using UAV hyperspectral data with spectral calibration and indices

**DOI:** 10.1016/j.isci.2026.116455

**Published:** 2026-06-25

**Authors:** Xiaohan Zhang, Yulan Tang, Qing-Wei Wang, Diannan Huang, Yue Feng, Jingli Wang, Zhao Wang, Yulong Cong

**Affiliations:** 1 School of Municipal and Environmental Engineering, Shenyang Jianzhu University, Shenyang 110168, China; 2 CAS Key Laboratory of Forest Ecology and Silviculture, Institute of Applied Ecology, Chinese Academy of Sciences, Shenyang 110016, China; 3 School of Transportation and Surveying Engineering, Shenyang Jianzhu University, Shenyang 110168, China; 4 Liaoning Inspection, Examination and Certification Centre, Shenyang 110032, China

**Keywords:** soil science, applied sciences, modeling in soil science

## Abstract

In this study, UAV hyperspectral imagery and 52 topsoil samples were collected from a representative industrial legacy site in Tieling City, Liaoning Province. UAV spectra were calibrated to lab spectra using piecewise direct standardization (PDS). Feature bands were selected using the competitive adaptive reweighted sampling (CARS) method; feature band combinations were created using the dual-band spectral indices (DBSIs) and three-band spectral indices (TBSIs). A Transformer algorithm was optimized by particle swarm optimization (PSO) to predict soil copper (Cu) and arsenic (As) and to generate distribution maps. The results indicated that the PDS substantially reduced environmental effects in the UAV data. Spectral indices improved prediction accuracy, and TBSIs consistently outperformed DBSIs. The PSO-Transformer with TBSI-3 achieved the best performance, with validation R^2^ of 0.83 for Cu and 0.88 for As. We propose a “sky-ground” hyperspectral inversion model. It enables high-accuracy prediction of soil heavy metal concentrations and provides a robust tool for monitoring contamination in industrial legacy sites.

## Highlights

• “Air-ground” synergy improves sensor consistency across lab and UAV environments
• A PSO-Transformer model was developed for accurate prediction of soil heavy metal content
• CARS and spectral index feature selection were integrated to evaluate model performance
• Full-site mapping of Cu and As contents across industrial legacy site was achieved

## Graphical abstract

**Figure:**
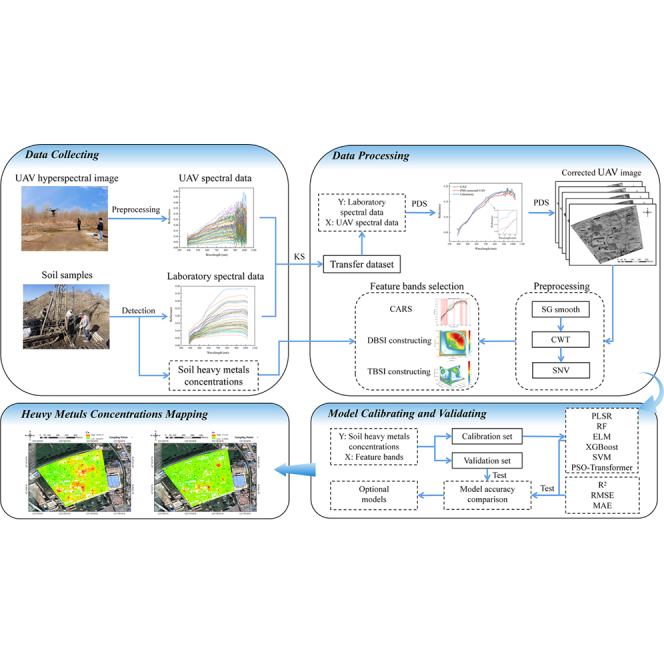

## Introduction

With the advancement of industrialization and urbanization, a large number of industrial sites have been relocated.^1^^,^^2^ Industrial enterprises, through historical industrial processes, such as casting, machining, and heat treatment, have led to elevated heavy metal levels in soils located in and surrounding industrial zones, surpassing regulatory thresholds.^3^ This has resulted in industrial legacy sites that may fail to meet the environmental standards required for government-designated land, thereby necessitating the initiation of contamination investigations for these parcels.^4^^,^^5^ Traditional soil monitoring methods rely on laboratory analysis of soil samples and interpolation to generate a heavy metal distribution map. Monitoring accuracy generally improves with increased sampling density, which poses a challenge for reliably capturing the spatial continuity of heavy metal distribution under sparse sampling conditions.^6^ Furthermore, this method is both labor and resource intensive, and the sampling process is vulnerable to causing secondary environmental pollution. Therefore, an efficient, accurate, and low-cost environmental monitoring method that also minimizes environmental impact during field investigations is urgently needed.

Hyperspectral imaging has emerged as an effective technique for regional-scale detection and quantification of heavy metal pollutants in soils.^7^^,^^8^ Hyperspectral imaging systems derive fine spectral and spatial characteristics of targets through the analysis of their reflected or emitted electromagnetic radiation across contiguous narrow bands.^9^ Current hyperspectral remote sensing platforms, including airborne as well as ground- and satellite-based technologies,^10^^,^^11^^,^^12^ have been widely used to monitor soil heavy metal contamination. Ground-based hyperspectral imaging, conducted *in situ* or in laboratories, provides high accuracy but is constrained by limited spatial coverage.^13^ Satellite-based hyperspectral imaging enables large-area monitoring with systems such as GF-5 and ZY-1-02D but suffers from low spatial resolution (10–100 m), leading to mixed-pixel effects in heterogeneous environments.^14^^,^^15^ However, although survey accuracy requirements are typically at the meter level in small-scale study areas such as industrial legacy sites, this resolution is insufficient to meet practical needs for accurate soil heavy metal inversion. Recent developments in UAV-based airborne hyperspectral systems provide high-resolution, flexible, and timely data acquisition, which makes them ideal for small-scale studies.^16^^,^^17^^,^^18^ UAV hyperspectral imaging can mitigate atmospheric interference and improve the mapping accuracy. Studies have shown its effectiveness in mining areas for detecting As and Cu.^19^^,^^20^ Most previous UAV-based hyperspectral studies on soil heavy metal inversion have focused on mining areas. However, in small-scale industrial legacy sites, the surface cover is more complex, rendering the inversion task more challenging. Therefore, in this study, the piecewise direct standardization (PDS) algorithm was applied after extracting the bare soil from the study area to correct the UAV spectral data using laboratory spectral data. This process mitigates the influence of environmental variations, thereby enhancing the precision of heavy metal content predictions.

Full-band inversion is often computationally expensive and prone to overfitting due to redundancy and environmental noise.^21^^,^^22^ Feature selection algorithms—such as competitive adaptive reweighted sampling (CARS), genetic algorithms, successive projection algorithm (SPA), and uninformative variable elimination (UVE)—are effective in reducing spectral dimensionality^23^^,^^24^ but may fail to capture trace-level indicators.^25^ In contrast, the spectral index method combines multiple bands through mathematical operations to enhance surface reflectance features.^26^^,^^27^ By constructing dual-band spectral indices (DBSIs) and three-band spectral indices (TBSIs), it reduces multicollinearity and redundancy while preserving key spectral information.^28^^,^^29^ However, studies on the use of the spectral index for selecting optimal characteristic band combinations for soil heavy metal analysis remain scarce. Moreover, the spectral index results vary under different environmental conditions, highlighting the need for further investigation into its applicability for heavy metal inversion in industrial site soils.

**Figure 1:**
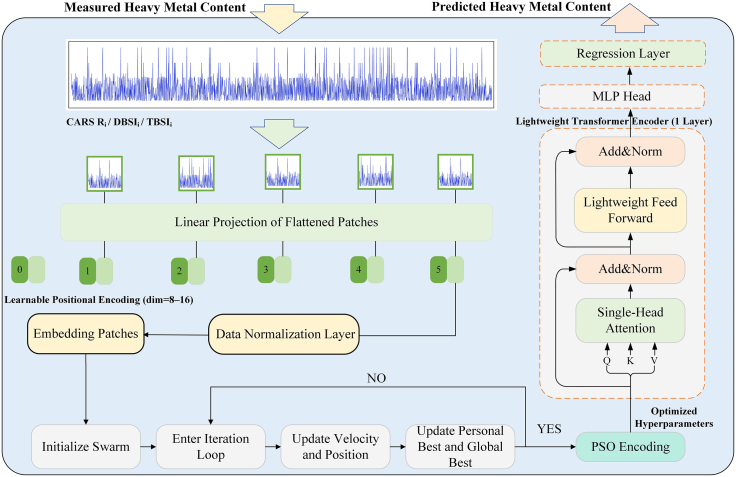
Flowchart of PSO-Transformer

In addition to selecting essential spectral bands or indices, choosing suitable models for inverse modeling is crucial. A model aims to establish specific relationships between spectral features and soil heavy metal content. Partial least-squares regression (PLSR) is widely used because it effectively addresses multicollinearity and small-sample constraints. However, with the increasing dimensionality and complexity of spectral data, its ability to capture nonlinear relationships and maintain robustness under high-noise conditions becomes limited.^30^ Random Forest (RF), as an ensemble learning approach, is well suited for hyperspectral applications due to its strong feature-selection capability and resistance to overfitting, and it has been shown to maintain stable accuracy, even with substantial missing data.^31^ Support Vector Machine (SVM) leverages kernel-based mapping to handle high-dimensional spectra with limited samples and consistently demonstrates strong generalization; when fewer than 50 samples are available, its inversion accuracy for soil heavy metals often exceeds 0.77.^32^^,^^33^ Extreme Learning Machine (ELM), with its simple architecture and fast training, provides an efficient solution for the rapid processing of large hyperspectral datasets. For example, Lu et al.^34^ modeled heavy metal concentrations using multiple preprocessing strategies and ELM for 100 soil samples collected from Guizhou Province, achieving the highest prediction performance for Cr and Cu with R^2^ values up to 0.88. Deep Neural Networks (DNNs) achieve good predictive performance (R^2^ = 0.59–0.92) but still risk overfitting and require improvement.^35^^,^^36^^,^^37^ To this end, we propose a PSO-Transformer model that leverages the Transformer architecture^38^ for its strength in modeling complex relationships (Figure 1). We employed the particle swarm optimization (PSO) algorithm to optimize key hyperparameters of the simplified Transformer model, including the initial learning rate, L2 regularization coefficient, and the number of training iterations. These optimizations ensured stable convergence of the lightweight single-head, single-layer Transformer, constrained model complexity under small-sample conditions, prevented overfitting, and enhanced the overall prediction performance.

**Figure 2:**
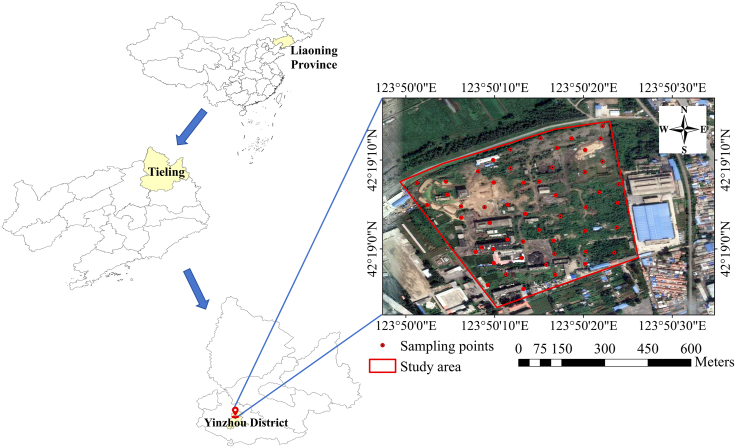
Overview of the study area

In this study, we selected an industrial legacy site in Tieling City, Northeast China, formerly the Tieling Non-ferrous Metal Processing Plant, as the study area (Figure 2). Historical records indicate that the plant operated copper smelting and production for more than 60 years, resulting in severe contamination by heavy metals, particularly copper (Cu) and arsenic (As). In summary, this study aimed to (1) construct an “air-ground” hyperspectral inversion model by implementing the PDS algorithm to conduct cross-environmental spectral calibration between UAV systems and laboratory-established reference baselines, effectively suppressing sensor drift attributable to environmental variability, and to improve the inversion accuracy over the whole region; (2) compare and analyze the CARS feature band selection algorithm as well as the DBSI and TBSI methods to further explore spectral information and enhance key spectral features; and (3) develop the PSO-Transformer model to extract potential information from hyperspectral data and improve model performance. Finally, distribution maps for Cu and As in the study area were developed to guide future soil remediation initiatives.

## Results

### Descriptive statistics of heavy metal content

The study area is a residential land in Tieling City Urban Master Plan (2014–2030); thus, the screening value of the first category of construction land in the Soil Environmental Quality Soil Pollution Risk Control Standard for Construction Land (GB 3600-2018) was selected. Owing to the lack of information on soil background values in Tieling City, the soil environmental background values in Xinchengzi District, Shenyang City, were selected as a reference. The descriptive statistical results of Cu and As are presented in Table 1.

**Table 1:** Statistical data on Cu and As contents (unit: mg/kg)

Element	Max	Min	Mean	SD	CV(%)	Background Value	Screening Value
Cu	7630.00	39.00	1609.08	1564.30	97.22	23.07	2000.00
As	417.00	3.61	41.89	62.19	148.47	9.28	20.00

From the mean values, both element samples exceeded the soil background values, with Cu content not exceeding the screening values and As content exceeding the screening values by more than 2-fold. This suggests that the study area is contaminated to a certain degree by heavy metals due to anthropogenic influences compared with the original soil background condition. The coefficient of variation measures the variability between samples at different spatial and temporal scales, providing insight into the level of anthropogenic impact on the environment. A coefficient of variation of less than 10% is considered to be a weak variation; 10%–30% is considered moderate variation; and >30% is considered strong variation.^39^ The coefficients of variation for both elements were much larger than 30%, suggesting that the soil heavy metals were subjected to large external disturbances and exhibited uneven distribution and obvious spatial heterogeneity. Preliminary assessment indicated that both Cu and As were present at different levels of contamination in the study plots and should therefore receive particular attention.

### Calibration of UAV imagery with the PDS algorithm

**Figure 3:**
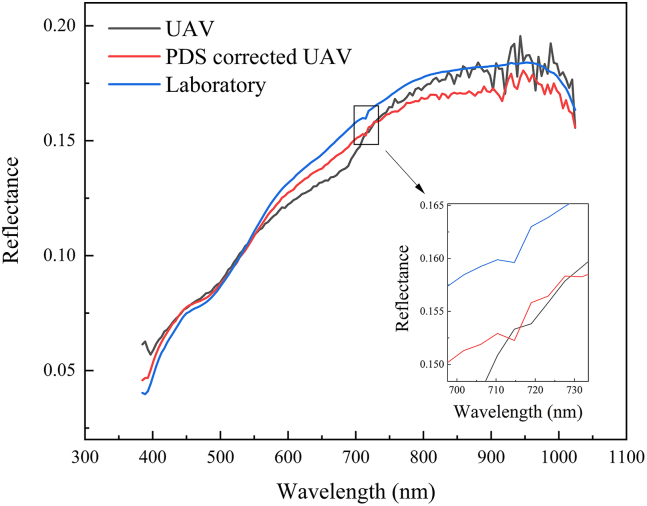
UAV spectral curves before and after PDS correction

The reflectance trends of UAV spectra, PDS-corrected UAV spectra, and laboratory spectra, each averaged across samples, are illustrated in Figure 3.

Although numerical differences were observed between the laboratory spectra and the uncorrected UAV spectra, they exhibited a generally consistent overall trend. The laboratory spectra presented a relatively smooth curve, whereas the UAV spectra contained more pronounced noise. This discrepancy was primarily attributed to atmospheric scattering during UAV data acquisition, with noticeable fluctuations near water absorption bands (e.g., around 950 nm). In addition, surface shadows, variations in viewing geometry, and differences in illumination conditions during UAV-based remote sensing further increased spectral noise. After PDS correction, the deviation between UAV and laboratory spectra was significantly reduced. The corrected UAV spectra exhibited slopes more consistent with those of the laboratory spectra in the 400–500 and 550–700 nm ranges and preserved key local spectral features such as the absorption trough near 715 nm. Overall, PDS correction improved the spectral consistency of UAV-derived data across multiple bands, mitigated the influence of environmental factors on spectral measurements, and provided a more robust basis for subsequent spectral feature selection and inversion modeling.

### Spectral feature selection

The PDS-corrected UAV data were smoothed using the Savitzky-Golay (SG) filter, followed by noise reduction through continuous wavelet transform (CWT), and then pre-processed with standard normal variable (SNV) transformation to highlight spectral data features. To extract important information from the spectral data, we selected the pre-processed spectral data for feature bands using CARS and constructed feature band combinations using DBSIs and TBSIs to prepare for the next step of inverse modeling.

### Selection of characteristic bands using CARS

On the basis of the number of spectral bands, we set the number of CARS iterations to 150^40^; the feature band selection results obtained from CARS are shown in Table 2.

**Table 2:** Feature band selection statistics based on the CARS

Element	Wavelength (Unit: nm)	Sum
Cu	388, 392, **396**, **413**, **421**, **425**, **429**, 453, **458**, 462, 466, 470, 478, 503, 507, 511, 515, 520, 524, 540, 545, 549, 688, 706, **714**, 854, **871**, 876, 880, **898**, **902**, 920, 925, **983**, 997, 1001, 1010, 1015, 1024	39
As	**396**, 400, 409, **413**, **421**, **425**, **429**, 433, 454, **458**, **714**, 718, 823, **871**, **898**, **902**, **983**, 992	18

The results suggested that the number of spectral bands was reduced from the original 150 to 39 for Cu and to 18 for As, thereby decreasing the modeling complexity. In addition, when comparing the characteristic band sets of the two heavy metal elements, the band selection algorithm identified several common bands (e.g., 413–429 and 898–902 nm). This suggests that the mechanisms by which Cu and As influence soil spectral reflectance are similar. In this study, the selected wavelengths were concentrated within three sensitive spectral regions. The 380–460 nm (blue-violet) region corresponds to Fe^3+^ electronic transition absorptions and is highly responsive to Cu/As interactions with iron (Fe) oxides. The repeated selection of bands such as 396, 413, 421, 425, and 429 nm indicates that Fe oxide-mediated changes are a major factor influencing the spectral response.^41^^,^^42^ The 680–720 nm (red-edge) region is associated with Fe oxide charge-transfer processes and red-edge slope variations, both of which are sensitive to metal-induced changes in mineral composition or organic-mineral complexation. The co-selected wavelengths (e.g., 714 nm) suggest that Cu and As affect oxidation states and mineral-organic associations in a manner that modifies the red-edge transition.^43^ Finally, the 820–1024 nm (NIR) region corresponds to overtone absorptions of O–H and metal–O bonds in clay minerals, with consistently selected wavelengths such as 871, 898, 902, and 983 nm reinforcing that the spectral variations primarily reflect the adsorption of Cu and As onto clay minerals and moisture-related vibrational features.^44^

### Selection of characteristic band combinations using DBSI methods

**Figure 4:**
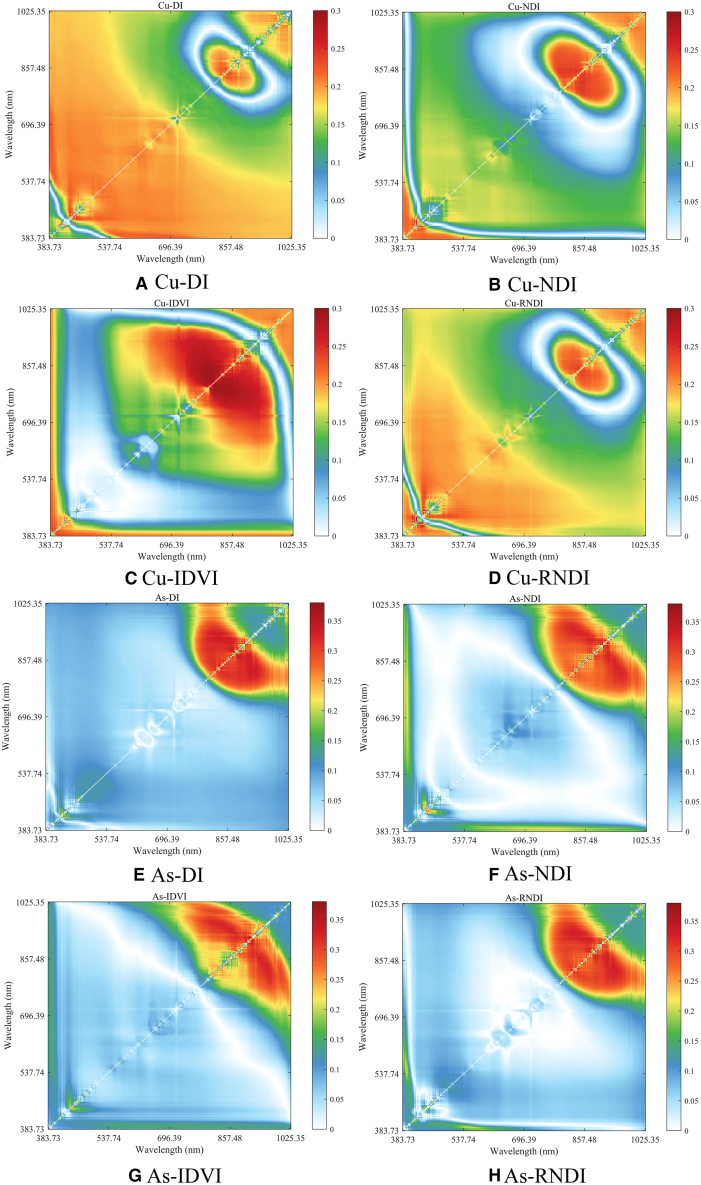
Heat maps of DBSI correlation with Cu and As contents (A–D) represent Cu-DI, Cu-NDI, Cu-IDVI, Cu-RNDI ,respectively. (E–H) represent As-DI, As-NDI, As-IDVI, As-RNDI, respectively.

Figure 4 presents the correlation coefficients of different DBSIs with Cu and As contents. For Cu, the sensitive bands of all four DBSIs were distributed within 769.84–864.10 nm, showing a strong correlation region, which suggests that these wavelengths are highly responsive to the Cu content. These wavelengths may align with Cu compound absorption peaks, implying their role in Cu content assessment. The inverse difference vegetation index (IDVI) had more combinations of bands with a strong correlation, and the most correlated sensitive bands were 800.37 and 811.31 nm, with a correlation coefficient of 0.303. Therefore, IDVI was considered as a method for constructing a DBSI for Cu. For As, the correlation characteristics of the four DBSI methods were more obvious, and the correlations of the characteristic band combinations were higher, with the maximum correlations of each spectral index ranging from 0.366 to 0.376. The sensitive bands of all four DBSIs distributed in the range of 824.46–919.61 nm showed a strong correlation region, which indicates that in this part of the wavelength interval, the As content has a specific spectral response. The difference index (DI) had more band combinations with a strong correlation, and the most correlated sensitive bands were 906.24 and 904.01 nm, with a correlation coefficient of 0.376. Therefore, we used DI as a method for constructing DBSI for As.

### Selection of characteristic band combinations using TBSI methods

**Figure 5:**
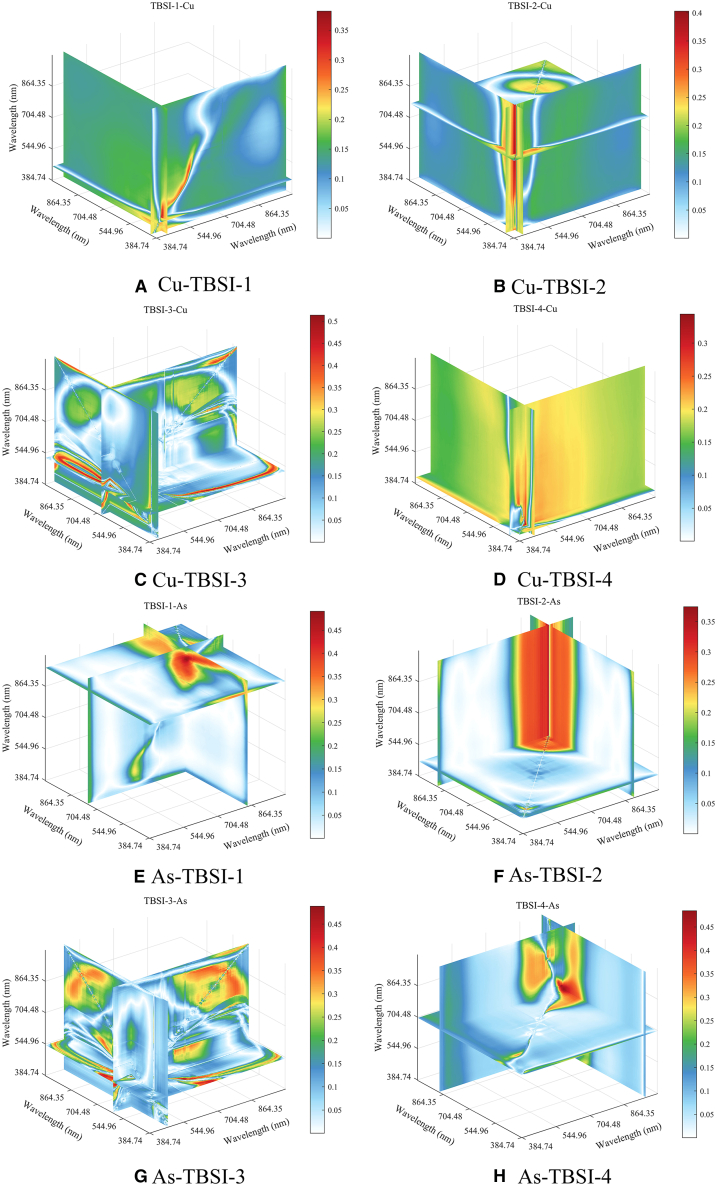
Heat maps of TBSI correlation with Cu and As contents (A–D) represent Cu-TBSI-1, Cu-TBSI-2, Cu-TBSI-3, Cu-TBSI-4, respectively. (E–H) represent As-TBSI-1, As-TBSI-2, As-TBSI-3, As-TBSI-4, respectively.

The correlation coefficients of the different TBSIs with the heavy metal content are presented in Figure 5. The spectral indices of Cu and As indicated the best correlation in TBSI-3. The correlation of Cu reached the highest of 0.514 with the best band combination of 422.31, 672.83, and 512.79 nm; the correlation of As reached up to 0.489 with the best band combination of 465.29, 600.65, and 531.49 nm. The band combinations in the region of maximum correlation in TBSI-3 were similar for both heavy metals, indicating that the mechanism of complex interaction of the Cu and As contents with the environment may be similar. Therefore, we used TBSI-3 to construct the TBSI of Cu and As.

### Regression model

**Figure 6:**
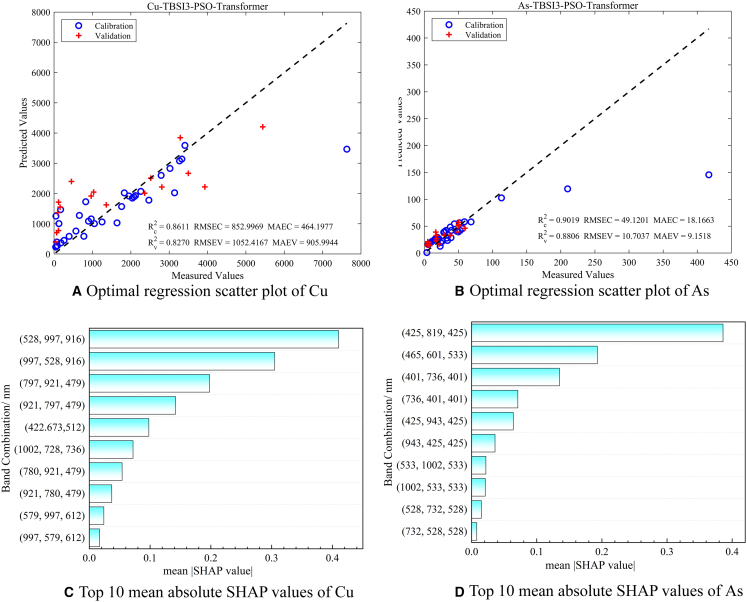
Best prediction results from different models (A) and (B) represent optimal regression scatter plots of Cu and As, respectively. (C) and (D) represent the top 10 mean absolute SHAP values of Cu and As, respectively.

We added six models for modeling and estimation, namely PLSR, RF, ELM, XGBoost, SVM, and PSO-Transformer. The frequency bands selected by CARS and the combination of bands selected using the DBSI and TBSI were incorporated into the model to ensure its accuracy. The training and validation sets were cross-validated; the computational results of the models are presented in Table S1, and the best prediction of Cu and As is demonstrated in Figure 6.

Overall, the predictive ability of the models for As was generally better than that for Cu, with *R*_*v*_^*2*^ ranging from 0.2080 to 0.8270 for the elemental Cu test set and from 0.3282 to 0.8806 for the elemental As. Root-mean-square error (RMSE) and the mean absolute error (MAE) values for Cu were excessively high, with RMSE_v_ ranging from 1285.6355 to 1963.7710 and MAE_v_ ranging from 1003.5293 to 1782.3563. This suggests that the model exhibited a large prediction error for certain regions or specific samples. Although the overall explained variance was high, RMSE and MAE remained large. RMSE and MAE are susceptible to the influence of outliers, and poor model performance on these anomalous points can significantly inflate prediction errors. It was due to differences in the datasets themselves. Combined with Table 1, it can be observed that the standard deviation for Cu is significantly larger (SD_Cu_ = 1,564.30 mg/kg, SD_As_ = 62.19 mg/kg), and the spatial heterogeneity of Cu is stronger in the study area, which might have led to poor model prediction performance. The PLSR model exhibited a mediocre performance, with the *R*^*2*^ values lower than 0.6 on the test set, showing poor predictive fitting. The RF and SVM models outperformed PLSR on the test set, with *R*^*2*^ on the test set reaching 0.6979 and 0.7912, respectively, which is a large improvement in prediction performance. The ELM model exhibited superior performance on the training set, with an *R*^*2*^ maximum value close to 0.9; however, ELM demonstrated weak generalization on the test set, suggesting that the model suffered from overfitting problems. The PSO-Transformer model exhibited superior predictive performance for both elements, with the highest Cu in the test set at 0.8270 and As at 0.8806. The model showed strong fitting ability on the training set while effectively adapting to new data in the test set, demonstrating good generalization capability. Although Cu in the CARS algorithm selected more characteristic bands, it did not enhance the prediction performance of the model. This suggests that the number of bands may not be directly related to model performance, and optimizing the selection of more accurate bands could prove more beneficial. The use of the spectral index method significantly improved the prediction performance of the model. Comparisons between the different metrics indicated that the TBSI method enhanced the accuracy of the model more than the DBSI method, consistent with the results of previous studies.^45^^,^^46^ The construction of spectral indices can incorporate more sensitive bands into the calculation of complex nonlinear relationships; however, the complex spectral response mechanism has not been quantitatively explained yet. Among the different combinations, the highest accuracy was obtained by combining TBSI-3 with PSO-Transformer, with this model combination demonstrating the enhanced predictive and generalization capabilities.

The Shapley Additive explanation (SHAP) analyses of the optimal Cu and As models (Figures 6C and 6D) showed that only a small subset of band combinations contributed substantially to the prediction accuracy, whereas most features exerted relatively minor effects. For Cu, the dominant contribution arose from the 528, 997, and 916 nm combination, linking the visible green region with two NIR wavelengths and indicating strong Cu-related spectral interactions. For As, the highest contribution was associated with the 425, 819, and 425 nm combination, involving blue and NIR bands that enhanced the weak indirect spectral response of As. In both models, the remaining combinations exhibited markedly lower SHAP values, illustrating that the PSO-Transformer concentrated its attention on a limited number of discriminative spectral interactions. When considered together with the optimal TBSI-3 correlation-based band combinations (Figures 5C and 5G), it became clear that the bands with the highest linear correlation coefficients did not necessarily correspond to the largest SHAP values. Correlation analyses capture pairwise linear association, whereas SHAP analyses quantify the marginal contribution of a feature within a nonlinear multivariate model. Consequently, a band combination could show strong correlation yet rank low in SHAP importance if its information was redundant or overshadowed by stronger nonlinear interactions learned by the model.

The residual plots of the optimal inversion models for Cu and As are shown in Figure S1. For Cu, the PSO-Transformer combined with TBSI-3 generated residuals that were generally centered around zero for both calibration and validation sets, indicating stable predictive behavior. Only a few high-concentration samples showed larger deviations, while no systematic overestimation or underestimation trend was observed. For As, the residuals were more tightly clustered, reflecting the higher accuracy achieved by the optimal model. Most samples exhibited small residuals within a narrow interval, and only several samples with higher As content displayed moderate prediction errors. The variance of residuals did not increase with concentration. Therefore, the PSO-Transformer models provided reliable predictions with minimal bias, supporting the robustness of the inversion results under small-sample conditions.

### Mapping of heavy metal content using UAV hyperspectral data

**Figure 7:**
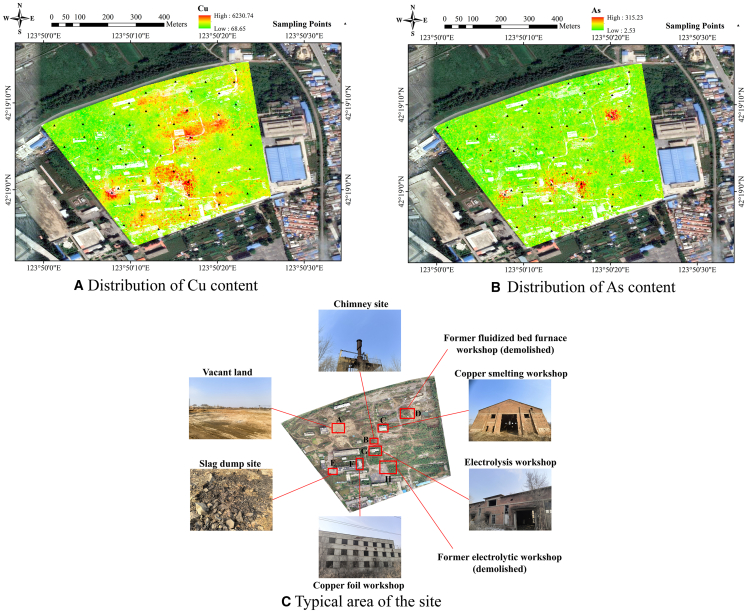
Distribution of soil heavy metal content in the study area (A) and (B) represent distribution of Cu and As content, respectively. (C) is typical area of the site.

In the previous section, we discuss the use of the PSO-Transformer model in combination with TBSI-3 to estimate soil Cu and As contents with high accuracy. The spectral reflectance of all pixel blocks in the UAV image was extracted to calculate the TBSI-3 values. Subsequently, the established PSO-Transformer model was used to invert the soil Cu and As contents for each pixel of the PDS-corrected UAV images. Finally, the distribution of the Cu and As contents in the soil was mapped (Figure 7).

As shown in Figures 7A and 7B, the Cu content varied from 68.65 to 6230.74 mg/kg, whereas the As content varied from 2.53 to 315.23 mg/kg, with Cu exhibiting greater extreme difference. Overall, the spatial distribution trends of the two heavy metals exhibited certain similarities, forming a pattern of sheet- and island-like distributions. The key industrial plants and roads within the study area showed varying degrees of contamination, with the most severely contaminated areas mainly located in Cu-refining, electrolysis, and Cu foil workshops (Figure 7C). The open area on the northwest side of the plant was cleaner, which is more consistent with the use of the land. In Cu-smelting and electrolysis processes, Cu and As contaminations are likely to occur due to improper management or pollution accidents, such as furnace leakage. Mining and smelting as well as the industrial waste and slag dumping of the non-ferrous metal processing plant are the primary sources of heavy metals at the site. The Cu-smelting process of the plant is pyrometallurgical smelting, and the melting and blowing slags produced in the process have high Cu content, which may cause pollution if not properly disposed of. Arsenic oxides are present in the flue gas produced by the Cu-smelting and converter-blowing processes.^47^^,^^48^ The chimney was located in the center of the plant, and northwest winds prevailed in the study area during winter; thus, it is assumed that As contamination in parts of the study area was likely influenced by atmospheric deposition from pollutant sources.

Therefore, the distribution maps generated using UAV hyperspectral imagery to predict the heavy metal content in soil from an industrial legacy site are effective. The distribution maps of soil heavy metal contamination generated using this method facilitate monitoring of heavy metal pollution and offer valuable guidance for the development of subsequent site remediation strategies. The research findings provide essential operational theories and technical methods for environmental monitoring of small-scale contaminated sites.

## Discussion

Soil spectral responses to heavy metals are complex due to various factors such as moisture, soil structure, and atmospheric interference. These variables complicate the direct assignment of specific spectral bands for metal quantification.^49^^,^^50^^,^^51^ The spectral signal strength obtained by ground-based hyperspectral sensors is optimal, and, therefore, the use of ground-based soil spectroscopy to assist satellite-based or airborne remote sensing platform technologies is necessary. Several researchers and scholars have employed the direct normalization (DS) algorithm to achieve the fusion of ground-based spectral data with satellite or airborne spectral data.^52^^,^^53^ The PDS algorithm is an improvement based on the DS algorithm, which is used for the fusion of ground-based spectral data with satellite-based or airborne spectral data by utilizing phase information for the spectral differences for optimization.^54^ The PDS algorithm employed in this study is less sensitive to spectral nonlinearities and more effectively utilizes the coherence feature of hyperspectral data, leading to reduced spectral noise interference and enhanced correction accuracy. In particular, it shows more stability and efficiency in processing complex or noisy hyperspectral data, weakening the influence of environmental factors on UAV spectral data. Moreover, the laboratory spectral data were obtained using a halogen lamp positioned at a 30° angle vertically, in accordance with a previous study.^55^ However, the acquisition of UAV-based hyperspectral imagery may be influenced by factors such as sensor positioning and illumination conditions (e.g., solar angle and light intensity), which were not accounted for in this study.

The CARS algorithm simplified the estimation model, and similar to other studies, Cu and As exhibited prominent response bands in the 413–429 nm visible and 871–902 nm near-infrared spectra.^56^ However, the characteristic spectral variables have differed across previous studies, indicating potential differences between datasets.^57^^,^^58^ For DBSI, the sensitive band of elemental Cu (769.84–864.10 nm) significantly overlapped with the charge transfer absorption band (800–900 nm) of Cu(II). This uptake resulted from electron transfer between the coordination layer of Cu(II) and hydroxyl (-OH) or water molecules in the soil, with its intensity regulated by soil pH and organic matter content.^59^^,^^60^ There were more combinations of bands highly correlated with the IDVI. IDVI is mainly used to assess the vegetation condition; its strong correlation in this wavelength range may indicate that Cu influences vegetation indirectly by altering soil chemistry. For As, the binding of As(III) with organic matter induced the overtone absorption of the As–O stretching vibration,^61^ forming a weak absorption feature in the near-infrared region (898–908 nm). This provided a theoretical basis for selecting band combinations in DBSI (824.46–919.61 nm). The superior performance of TBSI-3 can be explained by the combined effect of the soil-metal spectral mechanisms and the mathematical structure of the index. The characteristic bands identified for Cu and As were closely linked to known chemical interactions in the soil matrix. For Cu, the visible region (410–660 nm) and the 800–900 nm charge-transfer band reflected electron transitions associated with the humic substance chromophores and Cu(II) complexation. For As, the near-infrared overtone absorptions from As–O vibrations (898–908 nm) and ligand-to-metal charge transfer in As(V)-FeOOH complexes (550–600 nm) produced subtle but diagnostic spectral features.^62^^,^^63^ TBSI-3, formulated as (R_i −_ R_j_)/(R_i_ + R_j −_ 2R_k_), was particularly effective in enhancing these weak absorption signals. Its contrast term (R_i −_ R_j_) amplified metal-related reflectance differences, while the denominator suppressed background effects such as brightness and moisture variation. More importantly, the three-band structure captured nonlinear interactions arising from organic matter complexation and Fe-oxide binding. Consequently, TBSI-3 strengthened chemically meaningful spectral responses and provided a more discriminative input for the inversion models.

The PSO-Transformer demonstrated higher predictive accuracy than traditional machine-learning models because its architecture had been deliberately simplified and adapted to the limited data scale. Unlike the traditional Transformer model,^64^ the encoder-decoder structure was adjusted, leaving only a lightweight self-attention module with a narrow hidden dimension and reduced positional encoding, resulting in a parameter size comparable to or smaller than that of ELM or SVM. After dimensionality reduction through CARS or spectral index construction, the input space was substantially compressed, allowing the simplified Transformer to avoid overfitting while still capturing nonlinear spectral interactions more effectively than conventional algorithms.^65^^,^^66^ PSO further improved model performance by optimizing key hyperparameters such as learning rate, iteration number, and regularization strength, ensuring stable convergence and preventing local minima. With these combined adjustments, the lightweight PSO-Transformer maintained sufficient modeling capacity under small-sample conditions and achieved consistently superior accuracy and robustness for both Cu and As inversion.

### Conclusions and implications

This study proposes a comprehensive “sky-ground” hyperspectral inversion framework for mapping soil Cu and As concentrations by integrating laboratory spectroscopy, UAV hyperspectral imagery, and multistage spectral calibration.

#### Data and methodological innovations

Laboratory-UAV consistency was improved through PDS-based spectral calibration, enabling effective transfer of laboratory spectral characteristics to field-scale UAV observations. Feature dimensionality was optimized using CARS, while newly constructed spectral indices—particularly the TBSI series—enhanced the representation of heavy metal-related spectral responses. Furthermore, a lightweight PSO-Transformer model was designed to achieve stable nonlinear learning under small-sample conditions.

#### Core results

The PDS algorithm significantly improved spectral alignment between laboratory and UAV data. Although CARS achieved dimensionality reduction, spectral index-based modeling greatly outperformed raw spectral bands. TBSI-3, in particular, yielded substantial accuracy gains. Among all tested models, the PSO-Transformer demonstrated the highest predictive performance and robustness. The TBSI-3-PSO-Transformer combination achieved the best results for As (R^2^ = 0.8806, RMSE = 10.70, MAE = 9.15) and also improved Cu prediction compared with traditional machine learning models.

#### Application value

The integration of spectral index construction with an optimized deep learning model provides a reliable and scalable solution for high-precision soil heavy metal monitoring. The proposed framework offers practical value for environmental assessment, pollution risk mapping, and rapid investigation of contaminated sites using UAV hyperspectral platforms.

### Limitations of the study

The intrinsic mechanisms of the spectral response of soil heavy metals, particularly regarding the patterns of atomic energy and spectral band changes, were not fully explored in our study. Future studies should combine typical outdoor soil samples to establish mapping relationships between the absorption characteristics and reflectance spectra of soil heavy metals to explore the spectral response mechanisms of different soil heavy metals. This will require interdisciplinary collaboration among remote sensing, environmental science, and computer science, ultimately facilitating the development of a regional soil heavy metal spectral database to support accurate inversion of soil heavy metal content at the regional scale.

## Resource availability

### Lead contact

Requests for further information and resources should be directed to and will be fulfilled by the lead contact, Yulan Tang (tyl98037@sjzu.edu.cn).

### Materials availability

This study did not generate any new unique reagents, biological materials, microbial strains, cell lines, or other materials. Therefore, no materials are available for distribution. Detailed descriptions of the study area, soil sample collection procedures, laboratory hyperspectral measurements, and UAV hyperspectral image acquisition and processing are provided in the STAR Methods section to facilitate reproducibility of the study.

### Data and code availability

• Data: All data reported in this paper will be shared by the lead contact upon request.
• Code: All code reported in this paper will be shared by the lead contact upon request.
• Additional information: Any additional information required to reanalyze the data reported in this article is available from the lead contact upon request.

## Acknowledgments

We would like to express our sincere gratitude to the editors and reviewers who have put considerable time and effort into their comments on this article. This study was supported by the 10.13039/501100012166National Key Research and Development Program of China (no. 2018YFC1801200) and the Basic Research Project of Universities of Liaoning Province (Z2224002).

## Author contributions

Conceptualization, X.Z., Y.T., and D.H.; data curation, Z.W.; methodology, X.Z.; investigation, X.Z., D.H., Y.F., J.W., Z.W., and Y.C.; formal analysis, X.Z., Q.-W.W., Y.F., J.W., and Y.C.; writing – original draft, X.Z. and D.H.; writing – review & editing, Y.T. and Q.-W.W.; funding acquisition, X.Z. and Y.T.

## Declaration of interests

The authors declare no competing interests.

## STAR★Methods

### Key resources table

**Table:** 

REAGENT or RESOURCE	SOURCE	IDENTIFIER
Python	Python Software Foundation	Python 3.12.0
ArcGIS	Esri	ArcGIS 10.8
Origin	OriginLab	Origin 2024
MATLAB	MATLAB Software Foundation	Version R2023a
SPSS	IBM	IBM SPSS Statistics 26.0
ENVI	NV5 Geospatial	ENVI 6.2
MegaCube	IRIS Inc.	MegaCube V2.19.0
Omap	IRIS Inc.	Omap V2.0
sbgCenterNV5 Geospatial	SBG Systems	sbgCenter v7.1

### Method details

#### Workflow

The workflow of this study is presented in Figure 8, and the research methodology included five main steps: (1) The Cu and As contents and spectral reflectance in soil samples were measured, representative samples were selected using the Kennard-Stone (KS) algorithm and the UAV spectral data were corrected based on the laboratory spectral reflectance using the PDS algorithm. (2) The corrected spectral curves were smoothed using the Savitzky-Golay (SG) filter and then processed for noise reduction using continuous wavelet transform (CWT), followed by standard normal variable (SNV) transformation. (3) Feature bands were selected using the CARS algorithm. DBSI and TBSI were applied to construct feature band combinations to evaluate their effectiveness in improving model accuracy. (4) The Transformer model was optimised using the PSO algorithm, and its performance was compared with those of PLSR, RF, ELM, XGBoost and SVM; in addition, a predictive model for estimating the Cu and As contents in the study area was developed. Model accuracy and stability were assessed using the coefficient of determination (*R*^*2*^), root-mean-square error (RMSE) and mean absolute error (MAE). (5) The spatial distribution of the Cu and As contents in soil was mapped.

**Figure:**
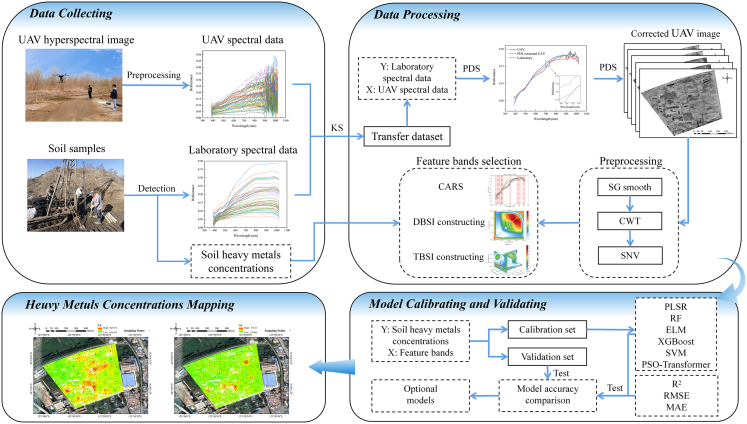
Flowchart of the study

#### Piecewise direct standardisation spectral correction algorithm

A linear relationship is established by PDS between the reflectance of the master and slave spectra.^67^ In particular, PDS can improve the measurement accuracy by analyzing the variation pattern between the laboratory and UAV spectra, thereby mitigating the impact of environmental factors, such as surface soil moisture, on the UAV spectrum. The PDS algorithm is expressed as follows:

(Equation 1) *X*_*Lab*(*m* × *n*)_ = *X*_*UAV*(*m* × *n*)_ × *β* + *E*

where *X*_*Lab (mxn)*_ denotes the laboratory spectral data of the transformed sample, with m denoting the number of transformed samples and n the number of spectral bands. The KS method is applied to identify the optimal number of transformed samples; *X*_*UAV (mxn)*_ represents the spectral data of the UAV-transformed samples; *β* is the transformation matrix used to quantify the difference between *X*_*Lab (mxn*)_ and *X*_*UAV (mxn)*_; and *E* is the residual matrix employed to correct the baseline bias arising from the difference between UAV and laboratory spectra.

The PDS transfer parameters were calculated by establishing a linear relationship between the laboratory spectra and UAV data for sample transfer. For each wavelength, laboratory absorbance values of the transfer samples were compared with the UAV spectral data at a specific wavelength and its adjacent counterpart within a designated window. The partial least-squares regression (PLSR) algorithm was used to model the relationship, and the PDS transformation matrix was generated by recording the regression coefficients on the spectra.^68^ Ultimately, the PDS parameters were used to normalise the UAV spectra from the validation dataset, enabling direct comparison with laboratory-measured field spectra and thereby mitigating environmental effects.

#### Characteristic bands and band combination selection

##### Competitive adaptive reweighted sampling

CARS is inspired by the principle of natural selection and effectively identifies optimal spectral-sensitive bands.^40^ First, the PLSR model is constructed by selecting some samples through Monte Carlo sampling, and the absolute value weights of reflectance in each band are calculated. Then, band variables with low regression coefficients are eliminated using an exponentially decreasing function. The selected subset of spectral bands is then cross-validated to identify the subset corresponding to the model with the lowest RMSE in the training set, representing the final set of selected sensitive bands.^69^ Assuming that the band reflectance data matrix of all samples is *X* and the target property matrix of all samples is *Y*; W is the combination coefficient of the data matrix; *T* is the score matrix of *X*, representing a linear combination of *X* and *W*; *c* is the regression coefficient of *Y* and *T* to construct the PLSR model; and *e* is the error vector, then we have

(Equation 2) *T* = *X* × *W*

(Equation 3) *Y* = *Tc* + *e* = *XWc* + *e* = *Xb* + *e*

where *b* is the combination of the combination coefficients and the regression coefficients and the weight *ω*_*i*_ is defined as

(Equation 4) $$\omega_{i}=\frac{\left|b_{i}\right|}{\sum_{i=1}^{n}\left|b_{i}\right|}$$

The lar*ger* |*b*_*i*_| and *ω*_*i*_ are in Equation 4, the more important the band variable is. Selecting a small number of sensitive bands using the CARS algorithm can effectively achieve dimensionality reduction, thereby improving the accuracy and efficiency of data modeling.

#### Construction of spectral indices

The spectral index method constructs new spectral index values by combining any two or more frequency bands.^70^ Pearson correlation analysis between the spectral index and heavy metal content was performed, and correlation coefficients were used to identify the optimal combination of frequency bands. Through band combination, a novel spectral index integrating multiple spectral bands was developed, effectively suppressing irrelevant spectral components such as background noise and atmospheric interferences. Reducing unnecessary data and highlighting the spectral characteristics of the target can improve the sensitivity of the spectral data to soil heavy metal content, which ultimately improves the performance of the inversion model. DBSI and TBSI used in this study are presented in Table S2. To ensure the inversion accuracy of the final model, we selected the band combinations with the correlation coefficient size in the top 10% for the final model construction.

#### Particle swarm optimisation Transformer (PSO-Transformer)

The Transformer model is a natural language processing model developed by Google researchers in 2017. It has been successfully applied by researchers and scholars to the prediction of atmospheric ozone or aerosol concentrations.^71^^,^^72^ This study considers Transformer architectures for soil heavy metal content prediction, inspired by the strong predictive performance of this. The Transformer model used in this study comprises input, location-embedding, self-attention, fully connected and regression layers. Among these, the self-attention layer is the core component of the Transformer model, which aims to capture contextual information by computing for each element of the input sequence a relationship with other elements in the sequence. The self-attention mechanism allows each input element to pay attention to other elements in the sequence as it is computed, thereby helping the model to understand the dependencies between the elements independent of the order of the sequence. The steps are described as follows:

**Step 1:** Input representation. The input sequence is first converted into a continuous dense vector representation by the embedding layer. This representation is an embedding vector for each word (or subword), which serves as the input for subsequent self-attention operations.

**Step 2:** Computation of *Q* (query), *K* (key) and *V* (value). To realise self-attention, the input vector is mapped into *Q*, *K* and *V*, respectively. This mapping is accomplished by three independent linear transformations (weight matrices). In particular, given a representation X of the input sequence, it is mapped as

(Equation 5) *Q* = *XW*_*Q*_, *K* = *XW*_*K*_, *V* = *XW*_*V*_

where the weight matrices *W*_*Q*_,*W*_*K*_ and *Wv* are parameters learned through training. With these mappings, each element of the input sequence has a new representation in the *Q*, *K* and *V* spaces.

**Step 3:** Calculation of attention weights. The self-attention mechanism obtains the attention weights by computing the similarity between queries and keys. In particular, for each *Q* and *K*, we compute their dot products and scale the results to avoid unstable values. The dot product results are then normalised using the softmax function to obtain the attention weight for each position. The attention weight *A* is computed as

(Equation 6) $$A=softmax\left(\frac{QK^{T}}{\sqrt{d_{k}}}\right)$$

where *d*_*k*_ denotes the dimension of the key vector; *K*^*T*^, the transpose of the key matrix; and *A*, the final attention weight matrix.

**Step 4:** Generation of weighted summation values. The final input indication for each position is obtained by weighted summation of the value matrix *V* with the computed attentional weight matrix *A. O* represents the weighted average of all input elements under the given query conditions and is an integration of the contextual information for each position in the input sequence.

**Step 5:** Attention mechanism. The standard multi-head structure was simplified to a single-head attention module to reduce model complexity and avoid overfitting under limited sample conditions. The query (Q), key (K), and value (V) vectors were projected into the same latent space, and self-attention was computed to capture the most informative spectral interactions relevant to heavy-metal prediction. The resulting attention output was then passed through a linear transformation to map it back to the required feature dimension. This lightweight design retained the core capability of attention to model long-range dependencies while ensuring suitability for small-sample spectral inversion.

**Step 6:** Output. The results after weighted summation are passed to the subsequent network layers as the outputs of the self-attention layer. These outputs represent the contextual information for each position in the input sequence and help capture the distantly dependent relationships in the sequence.

The performance of Transformer-based models was influenced by several key hyperparameters such as the learning rate and the strength of regularisation.^73^ In this study, the Transformer architecture was intentionally simplified to a lightweight configuration (one encoder layer and a single attention head) to ensure suitability under limited sample conditions. Even with this reduced complexity, selecting appropriate hyperparameters remained essential for achieving stable optimisation and preventing overfitting.The PSO algorithm was therefore employed to identify optimal hyperparameter values.The PSO algorithm was therefore employed to identify optimal hyperparameter values. PSO is a heuristic optimisation technique inspired by flocking behavior, in which particles iteratively update their positions and velocities based on their own best performance and the global optimum. Through such coordinated searching, the algorithm efficiently located well-balanced hyperparameter combinations that improved model convergence behavior.^74^ In this work, PSO was used to optimise the initial learning rate, the L2 regularisation coefficient, and the number of training iterations of the lightweight Transformer. These optimised settings helped stabilise training, reduce the risk of overfitting inherent to small datasets, and enhance the overall predictive performance. The flow of PSO-Transformer is shown in Figure 1. Furthermore, PLSR,^68^ RF,^75^ ELM,^76^ XGBoost,^22^ and SVM^32^ were adopted to assess the differences in model performance compared to the PSO-Transformer. Based on previous literature review and multiple experimental trials, the value ranges of the inversion model parameters are summarized in Table S3.

#### Model evaluation

We evaluated the model’s fitting and generalisation capabilities using three metrics, namely the coefficient of determination (*R*^*2*^), RMSE and MAE, which are expressed as follows:

(Equation 7) $$R^{2}=\frac{\sum_{i=1}^{n}{\left(\hat{y_{i}}-\bar{y}\right)}^{2}}{\sum_{i=1}^{n}{\left(y_{i}-\bar{y}\right)}^{2}}$$

(Equation 8) $$RMSE=\sqrt{\frac{\sum_{i=1}^{n}{\left(y_{i}-\hat{{\;y}_{i}}\right)}^{2}}{n}}$$

(Equation 9) $$MAE=\frac{\sum_{i=1}^{n}\left|y_{i}-\hat{{\;y}_{i}}\right|}{n}$$

where *y*_*i*_ denotes the true value of soil heavy metal content; $\bar{y}$, the average of the true value of soil heavy metal content; and $\hat{y}$, the predicted value of soil heavy metal content. *R*^*2*^ is used to reflect the goodness of fit of the inversion model to the data; the closer *R*^*2*^ is to 1, the more reliable the inversion model is. When the model prediction error is larger than the error calculated from the mean value, *R*^*2*^ is negative, indicating that the inversion model is unreliable. RMSE and MAE are employed to describe the error between the model prediction and the real value of metal content, where RMSE is employed to measure the dispersion of the error value. Low MAE and RMSE values indicate that the model is more reliable and the prediction is better.^77^

### Quantification and statistical analysis

This study employed Microsoft Excel and IBM SPSS Statistics 26.0 to perform descriptive statistical analysis of soil Cu and As concentrations, including mean, standard deviation, coefficient of variation, and comparisons with soil background and screening values (Table 1). Origin 2024, MATLAB R2023a and Python 3.12.0 were used to visualize spectral curves before and after PDS correction, correlation heat maps of dual-band and three-band spectral indices, and model prediction results (Figures 2, 6, and display figure). ArcGIS 10.8, ENVI, and MegaCube were used for UAV hyperspectral image preprocessing, georeferencing, mosaicking, hyperspectral data cube construction, and spatial mapping of predicted soil Cu and As concentrations across the industrial legacy site (Figure 7).
