# Supplementary material for: PSO-Transformer for mapping soil heavy metals using UAV hyperspectral data with spectral calibration and indices
Source: iScience. 2026 Jun 25;29(7):116455. doi: 10.1016/j.isci.2026.116455 (PMC13320560; doi:10.1016/j.isci.2026.116455)
Supplement: Document S1. Figure S1 and Tables S1–S3 [file mmc1.pdf]

**iScience, Volume *29***

## Supplemental information

# PSO-Transformer for mapping soil heavy metals using UAV hyperspectral data with spectral calibration and indices

**Xiaohan Zhang, Yulan Tang, Qing-Wei Wang, Diannan Huang, Yue Feng, Jingli Wang, Zhao Wang, and Yulong Cong**

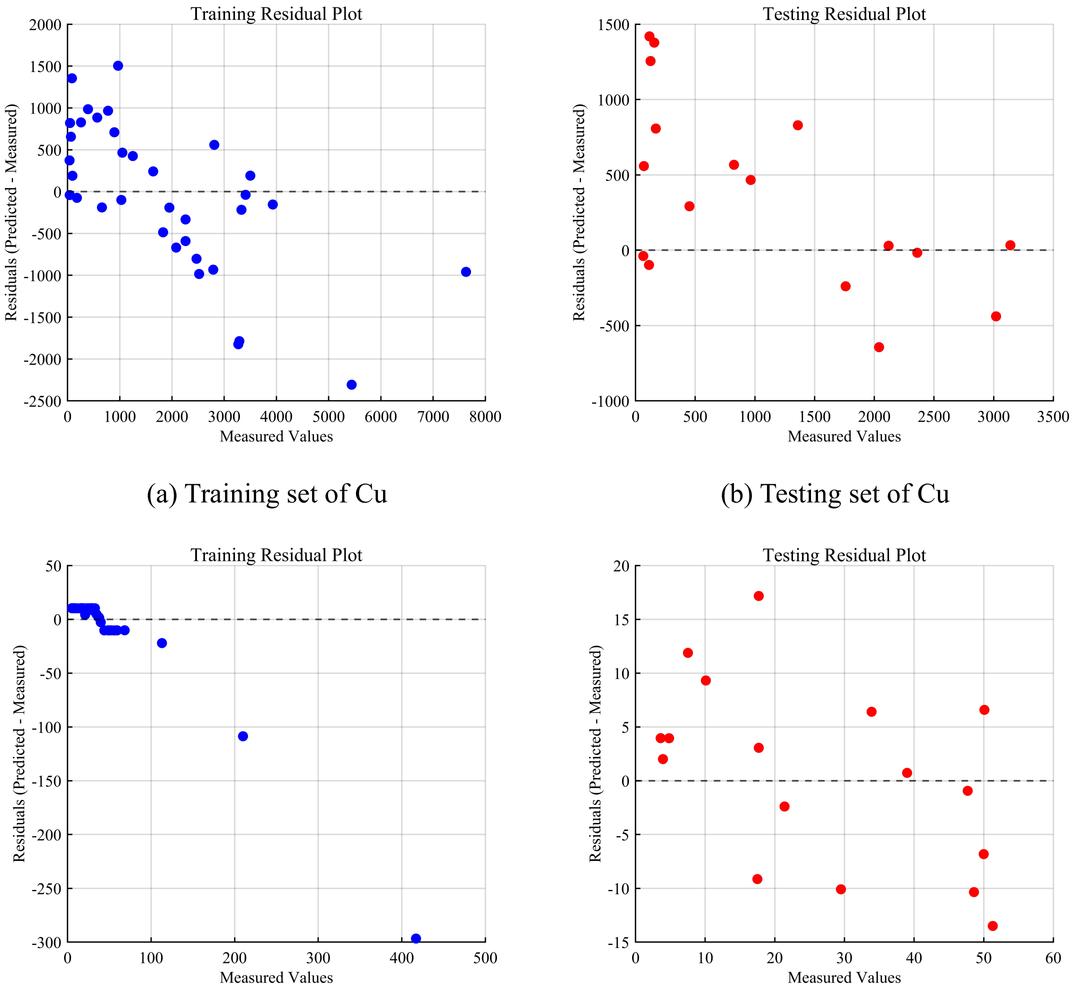


(a) Training set of Cu

(b) Testing set of Cu

(c) Training set of As

(d) Testing set of As

**Figure S1** Residual analysis plots of Cu and As.

**Table S1**

Model inversion results for all programs.

| Element | Model | Bands selection method | Training set | | | Testing set | | |
|---|---|---|---|---|---|---|---|---|
| | | | $R^2_c$ | $RMSE_c$ | $MAE_c$ | $R^2_v$ | $RMSE_v$ | $MAE_v$ |
| Cu | PLSR | CARS | 0.4236 | 1362.5685 | 836.6889 | 0.2362 | 1285.6355 | 1003.5293 |
| | | IDVI | 0.5807 | 1228.6361 | 721.8319 | 0.3554 | 1541.3031 | 1326.6570 |
| | | TBSI-3 | 0.4236 | 1396.4748 | 769.3566 | 0.3083 | 1663.7290 | 1251.5690 |
| | RF | CARS | 0.4240 | 1536.6855 | 793.6082 | 0.4119 | 1652.8927 | 1183.6928 |
| | | IDVI | 0.8190 | 944.1970 | 558.3249 | 0.6511 | 1277.4736 | 1042.6120 |
| | | TBSI-3 | 0.7125 | 1136.5661 | 736.2593 | 0.6390 | 1525.2593 | 1040.9072 |
| | ELM | CARS | 0.8639 | 753.5260 | 200.9934 | 0.2591 | 1963.7710 | 1782.3563 |
| | | IDVI | 0.8601 | 792.5645 | 157.6456 | 0.3914 | 1635.6568 | 1382.6785 |
| | | TBSI-3 | 0.8936 | 731.0299 | 176.6182 | 0.4460 | 1560.2545 | 1266.4864 |
| | XGBoost | CARS | 0.4823 | 833.6692 | 639.7422 | 0.2080 | 1693.6649 | 1539.6465 |
| | | IDVI | 0.5263 | 1305.9204 | 586.9570 | 0.3481 | 1596.4566 | 1440.9272 |
| | | TBSI-3 | 0.5155 | 1296.0580 | 738.0678 | 0.3724 | 1587.6679 | 1359.9962 |
| | SVM | CARS | 0.5836 | 1272.0036 | 636.8921 | 0.5870 | 1493.6828 | 1469.8265 |
| | | IDVI | 0.8256 | 936.9590 | 183.6459 | 0.7537 | 963.6540 | 1168.3891 |
| | | TBSI-3 | 0.7983 | 937.7080 | 384.6765 | 0.7912 | 1102.0426 | 886.2341 |
| | PSO-Transformer | CARS | 0.5936 | 1239.0285 | 723.5936 | 0.5271 | 1311.7305 | 1453.6238 |
| | | IDVI | 0.6826 | 1080.0527 | 708.9388 | 0.6250 | 1508.9360 | 1163.6802 |
| | | TBSI-3 | 0.8611 | 852.9969 | 464.1977 | **0.8270** | 1052.4167 | 905.9944 |
| As | PLSR | CARS | 0.4926 | 93.6355 | 34.6210 | 0.5039 | 31.6366 | 35.8758 |
| | | DI | 0.6614 | 65.7123 | 27.1582 | 0.5697 | 19.2776 | 17.5082 |
| | | TBSI-3 | 0.6399 | 73.9821 | 40.1136 | 0.5636 | 20.9355 | 15.4599 |
| | RF | CARS | 0.4366 | 146.3622 | 93.3641 | 0.3282 | 189.6380 | 65.9635 |
| | | DI | 0.6825 | 83.6220 | 50.1441 | 0.6929 | 20.9675 | 16.6889 |
| | | TBSI-3 | 0.8058 | 54.1844 | 22.8662 | 0.6967 | 13.4250 | 9.4591 |
| | ELM | CARS | 0.6625 | 82.0330 | 86.9941 | 0.3725 | 70.6693 | 63.2696 |
| | | DI | 0.7092 | 63.7993 | 24.3788 | 0.5195 | 21.8031 | 16.3465 |
| | | TBSI-3 | 0.7886 | 43.6605 | 20.5851 | 0.4236 | 56.2565 | 55.6829 |
| | XGBoost | CARS | 0.3663 | 65.9523 | 78.3599 | 0.3823 | 48.8556 | 38.5962 |
| | | DI | 0.4366 | 60.3655 | 56.9652 | 0.4240 | 36.3645 | 42.9986 |
| | | TBSI-3 | 0.6747 | 60.5280 | 27.3390 | 0.6481 | 18.4981 | 16.3331 |
| | SVM | CARS | 0.6329 | 65.3256 | 39.6833 | 0.6130 | 36.9680 | 18.0185 |
| | | DI | 0.8636 | 38.6566 | 30.0087 | 0.6725 | 19.6990 | 22.3644 |
| | | TBSI-3 | 0.8421 | 58.2912 | 26.6911 | 0.7270 | 14.8214 | 12.3343 |
| | PSO-Transformer | CARS | 0.7899 | 36.9563 | 20.9366 | 0.7125 | 12.3655 | 10.6850 |
| | | DI | 0.8736 | 53.6394 | 23.3361 | 0.8069 | 9.3955 | 10.2360 |
| | | TBSI-3 | 0.9019 | 49.1201 | 18.1663 | **0.8806** | 10.7037 | 9.1518 |

**Table S2**

Spectral index construction methods.

| | Spectral index | Construction form | Reference |
|---|---|---|---|
| DBSI | Difference Index (DI) | $R_i - R_j$ | [45] |
| | Inverse Difference Vegetation Index (IDVI) | $(1/R_i) - (1/R_j)$ | [46] |
| | Normalized Difference Index (NDI) | $(R_i - R_j)/(R_i + R_j)$ | [47] |
| | Re-normalized Difference Index (RNDI) | $(R_i - R_j)/\sqrt{(R_i + R_j)}$ | [48] |
| TBSI | TBSI-1 | $R_i/(R_j + R_k)$ | [49] |
| | TBSI-2 | $(R_i - R_j)/(R_i + R_k)$ | [29] |
| | TBSI-3 | $(R_i - R_j)/(R_i + R_j - 2R_k)$ | [50] |
| | TBSI-4 | $((R_i - R_j) - 0.2(R_i - R_k)) \times R_i/R_j$ | [51] |

Note. R is the value of spectrum, and the subscripts (i, j, and k) are the wavelengths.

**Table S3**

Range of parameter values used in the inversion model

| Model | Parameter | Range |
|---|---|---|
| PLSR | n-components | 2-40 |
| RF | Number of trees | 50-200 |
| | Depth | 3-15 |
| ELM | Batch | 10-60 |
| | Number of neurons | 1-60 |
| | Activation function | sigm/tanh/relu |
| XGBoost | Learning rate | 0.001-0.05 |
| | Number of trees | 1-200 |
| | Depth | 3-15 |
| SVM | Kernel type | RBF |
| | C | 0.01 – 1000 |
| | Gamma | 0.001 – 10 |
| PSO-Transformer | Number of particles | 30-50 |
| | Number of iterations | 80-150 |
| | Initial learning rate | 0.001-0.05 |
| | Number of attention mechanism heads | 1 |
| | L2 regularisation coefficients | 0.0001-0.01 |
